# Combined 3D Printed Template to Guide Iliosacral Screw Insertion for Sacral Fracture and Dislocation: A Retrospective Analysis

**DOI:** 10.1111/os.12620

**Published:** 2020-02-19

**Authors:** Chao Wu, Jia‐yan Deng, Tao Li, Lun Tan, De‐chao Yuan

**Affiliations:** ^1^ Orthopedics Center of Zigong Fourth People's Hospital Zigong China; ^2^ Digital Medical Center of Zigong Fourth People's Hospital Zigong China

**Keywords:** Combined template, Iliosacral screw, Minimally invasive, Sacral fracture, 3D printing technology

## Abstract

**Objective:**

To evaluate the accuracy and safety of a combined 3D printed guide template (combined template) to assist iliosacral (IS) screw placement for sacral fracture and dislocation.

**Methods:**

A total of 37 patients, 24 men and 13 women, age from 22 to 68 years old, diagnosed with a sacral fracture and dislocation were involved in this study for retrospective analysis from January 2016 to February 2018. There were 19 patients in the template group (42 screws) and 18 patients in the conventional group (31 screws). In the combined template group, IS screw placement was assisted by a combined 3D printed template; in the conventional group, the IS screws were placed freehand under fluoroscopy. The accuracy of the IS screw placement was evaluated by comparing the screw angle and the location of the screw entry point between the actual and the simulated screw in the combined template group. The safety of the IS screw placement was evaluated by comparing the quality of the reduction, the grading of the screws, the operation time, and radiation exposure times between groups.

**Results:**

A total of 73 pedicle screws were placed in 37 patients: 42 screws (30 S1, 12 S2) in the combined template group and 31 screws (23 S1, 8 S2) in the conventional group. In the conventional group, 1 patient developed symptoms of L5 nerve stimulation. In the combined template group, the average operative time of each screw was 25.01 ± 2.90 min, with average radiation exposure times of 12.05 ± 4.00. In the conventional group, the average operative time of each screw was 46.24 ± 9.59 min, with an average radiation exposure time of 56.10 ± 6.75. There were significant differences in operation and radiation exposure times between groups. The rate of screw perforation was lower in the combined template group (2 of 42 screws, 0 at grade III and 2 at grade II) than in the conventional group (5 of 38 screws, 2 at grade III and 3 at grade III). In the combined template group, the mean distance between the entry points of the actual and simulated screws was 1.4 ± 0.9 mm, with a mean angle of deviation of 2.1° ± 1.6°. All patients were followed up once every 3 months and were followed for 3 to 12 months.

**Conclusion:**

Using the combined template to assist with the insertion of IS screws delivered good accuracy, less fluoroscopy and shorter operation time, and avoided neurovascular injury as a result of screw malposition.

## Introduction

The sacrum is located between the spine and the pelvis and carries most of the weight of the body. The sacrum is a key part of the posterior pelvic ring^1^, ^2^, and maintains the stability of the pelvis^3^, ^4^. Unstable pelvic fractures are associated with increased risk of death^5^. However, 23% to 45% of pelvic ring fractures occur in the sacrum, which are difficult to diagnose and treat. Tile suggested that non‐surgical treatment was preferred for sacral fractures that did not damage the pelvic ring or break the stability of the lumbosacral joint^6^. However, it is generally accepted that a sacral fracture displacement of more than 10 mm or a sacral fracture displacement of 10 mm or less but with dislocation in the iliosacral (IS) joint can cause an unstable pelvic ring, thus necessitating surgery. In 1978, Letournel first reported the treatment of sacral fractures and dislocation through posterior open reduction with IS screw fixation^7^. Percutaneous IS screw fixation has been widely used in the clinic since it was first applied by Matta and Saucedo in 1989^8^. IS screw fixation can lessen tissue disruption, decrease the volume of blood loss, shorten operation time, and reduce infection rates, but there is a higher incidence of nerve and blood vessel injury and a higher radiation dose is required^9^. Accurate placement of the guide wire is key to performing this technique^10^.

Computer‐assisted screw placement can reduce the radiation dose used and improve the accuracy of screw placement^11^, but the long‐term learning curve and its high cost limit its application. A 3D printed navigation template can improve the accuracy of screw placement^12^; the long guide tube of the traditional 3D printed navigation template will increase the tension of the skin and soft tissue, which will affect the accuracy of screws; a large incision is generally needed to solve this problem, but causes great trauma. Furthermore, it is difficult to remove the guide template after inserting two or more guide needles. However, with only a single screw, the mechanical stability of the internal fixation is poor. Therefore, we developed a combined guide template for IS screws, with a base and guide pipe designed to be detachable and assembled at the site of the incisions. The guide template can be removed after K‐wire insertion and screw placement. This guide template reduces the trauma caused by traditional multiple‐screw placement and improves mechanical stability through multiple‐screw placement. In this study, the accuracy and safety of the combined navigation template were evaluated by comparing them with perspective guidance nailing technology.

The aims of this study included: (i) to explore the feasibility of the combined template assisting the IS screw insertion; (ii) to research the insertion accuracy of IS screws assisted by the combined template; and (iii) to investigate whether the combined template can improve the safety of IS screws in the treatment of sacral fracture and dislocation.

## Materials and Methods

### *General Information*

#### *Participants*

The inclusion criteria were: (i) Denis *et al*.^13^ classification of sacral fracture: area I or area II; (ii) Tile type B and C pelvic fractures with closed reduction; (iii) age over 18 years old; and (iv) closed fracture with an injury time of less than 3 weeks. The exclusion criteria were: (i) patients with dysfunction in at least one vital organ; (ii) fracture comminuted around the screw insertion point; and (iii) serious osteoporosis.

A total of 37 patients, 24 men and 13 women, aged from 22 to 68 years old, diagnosed with a sacral fracture and dislocation were involved in this study for retrospective analysis from January 2016 to February 2018. All patients were informed of the experimental design before the surgery and signed an informed consent form. This study was approved by the medical ethics committee of our hospital.

#### *Intervention*

The IS screw insertion was assisted by the combined templates.

#### *Comparison*

The IS screws were inserted under conventional fluoroscopy.

#### *Outcome*

The operation time and radiation exposure times were recorded. The quality of the reduction and grading criteria were evaluated, and the difference between actual and simulated screw positions in the combined template group were calculated.

#### *Study Design*

The present study is a case control study.

### *Template Design and Printing*

All patients underwent CT before surgery for template design and after surgery for assessment of screw placement. The thickness of the CT was 0.625 mm. The CT data were saved in Digital Imaging and Communications in Medicine (DICOM) format for transfer and template design.

**Figure 1:**
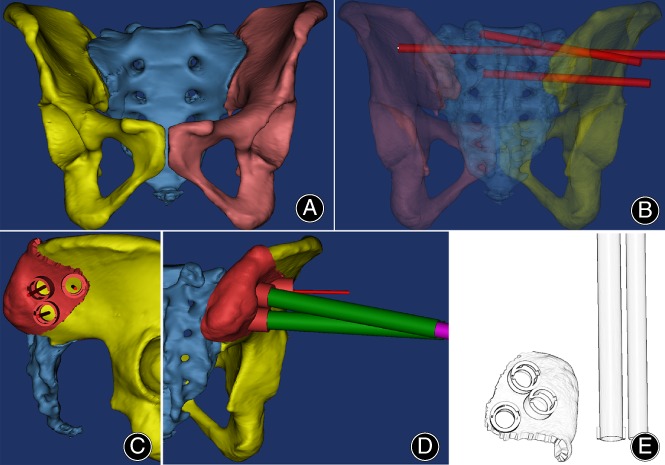
Novel template designed using software: (A) A 3D model of the pelvis was reconstructed; (B) simulation of implanting the iliosacral (IS) screw *via* S1 and S2 without any penetration; (C, D) the designed IS screw navigation template; and (E) a sketch of the novel template.

The CT data were imported into MIMICS 21.0 (Materialize, Leuven, Belgium) for 3D reconstruction of the pelvis and nailing design. First, the fracture was repositioned to a standardized anatomical structure

through simulation (Fig. 1A). Virtual screws (diameter of 6.5 mm) were placed in the ideal S1 and S2 trajectories of the 3D pelvis model. The virtual screws were placed such that they penetrated the cortex on the coronal, sagittal, and axial views (Fig. 1B). The 3D model of the pelvis and virtual screws were imported into 3‐matic 13.0 (Materialize, Leuven, Belgium). The pelvis was used as the substrate of the virtual template. The virtual screws were used to determine the direction and inner diameter of the template's guide pipes. The substrate and guide pipes of the template were designed to be detachable; inner pipes were placed inside the outer pipes, which were assembled into a whole by rotating. (Fig. 1C–E). The virtual templates were exported into STL format and then imported into 3D slicing software (accuracy, 0.014 mm; material, photosensitive resin) for printing (3DS, Projet 3600, United States). The virtual pelvis was imported into 3D slicing software (accuracy, 0.1 mm; material, PLA+) for printing (3D Talk, FAB 460, China).

In our research, a total of 8 h was needed for template design and printing, and a total of 24 h was needed for pelvis printing; the whole procedure took 26–32 h. The cost of the printed material for the template was approximately US$80 and the cost of the printed material for the pelvis was approximately US$100. The entire procedure for this study was completed at our hospital.

### *Preoperative Preparation*

**Figure 2:**
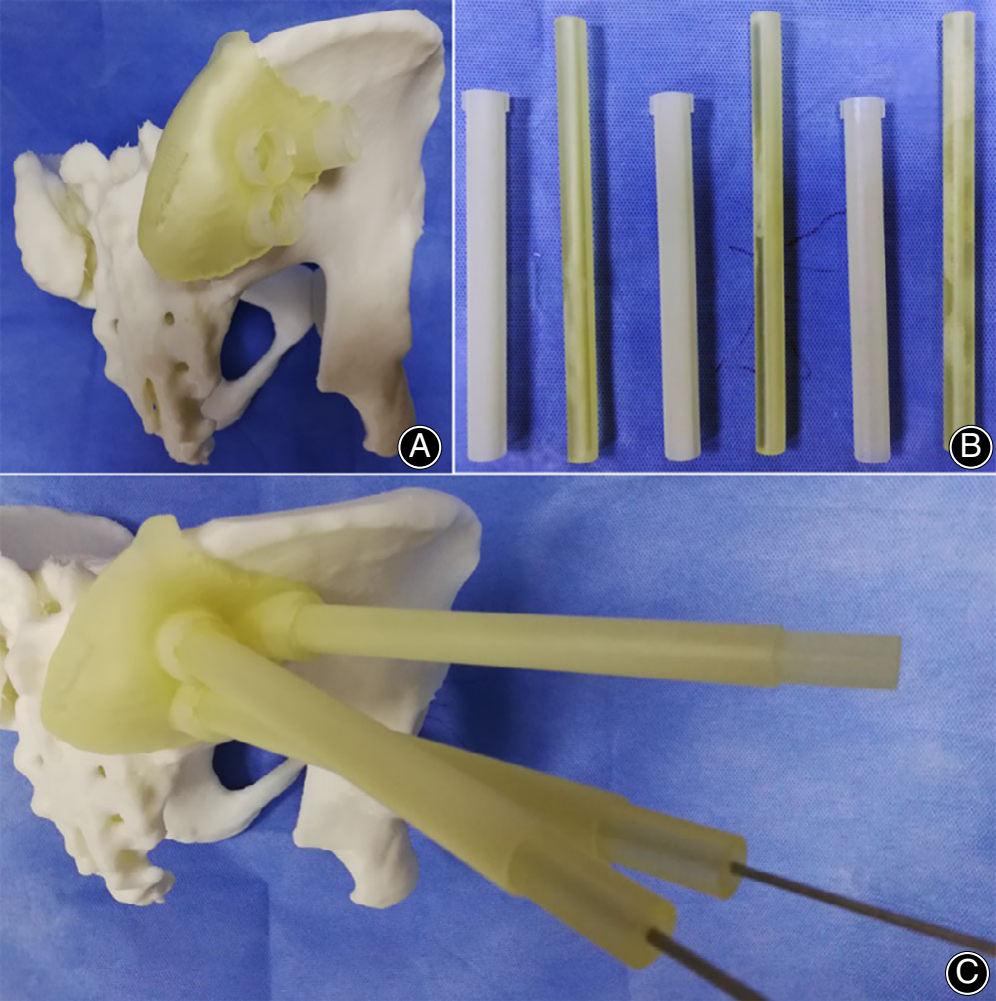
Preoperative application of the novel template: (A) The guiding template is attached to the iliac crest on the model; (B) the printed inner and outer pipes; and (C) the inner and outer sleeves mounted on the base, with K‐wire inserted into the model.

Preoperative treatment of other combined injuries: For type C fractures, the vertical displacement was essentially corrected by lower limb traction before the operation. The combined template was attached to the posterior superior iliac spine on the model. A K‐wire with a diameter of 2.5 mm was inserted through the inner pipe of the combined template to observe whether the route of the K‐wire was consistent with the preoperative design (Fig. 2).

### *Surgical technique*

All surgeries were performed by the same experienced surgeon.

Combined template group: All combined templates were sterilized with low‐temperature plasma.

Anesthesia and position: Under general anesthesia, the patient was placed in the prone position on a radiolucent operation table. Lower limb traction, Schanz nail lifting, and lateral extrusion of the crown rod were used for reduction of the posterior pelvic ring fracture or dislocation. The reduction was confirmed by fluoroscopy of the C‐arm, and traction or temporary fixation with a Kirschner wire was maintained.

Approach and exposure: The surface projection of the entry point on the skin was marked. An incision, approximately 5 cm, was made, and subcutaneous fascia and muscles were carefully stripped from the bones.

**Figure 3:**
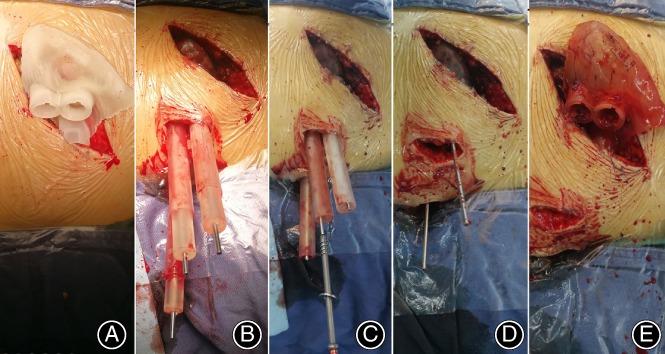
Intraoperative application of the novel template: (A) An incision (approximately 5 cm) was made, and muscles and fascia stripped from the iliac crest to insert the base of the template; (B) an incision (approximately 2 cm) was made at the location of the screw entry point, through which the inner pipes and outer pipes were inserted and mounted on the base, and one to three 2.5‐mm K‐wires were inserted into the planned inner pipes; (C) the inner pipes were removed and a 6.5‐mm cannulated screw was inserted, guided by the K‐wire; and (D, E) the outer pipes and template base were removed.

Fixation or placement of prosthesis: The sterilized pedestal of the template was inserted through the incision to make the template adhere to the bone completely (Fig. 3A). An incision of approximately 2 to 3 cm was made at the location of the screw entry point, through which the inner and outer pipes were inserted and mounted on the base (Fig. 3B).

Reconstruction: Depending on the fracture of the patient, between 1 and 3 K‐wires were inserted into the planned inner pipe guided by the combined template as a reference. Inlet and outlet fluoroscopy views were obtained to confirm the position of the K‐wires with minimum radiation exposure. The inner pipes were removed, and a cannulated screw with a diameter of 6.5 mm was then inserted, following the K‐wires (Fig. 3C). The outer pipes and template base were then removed (Fig. 3D,E). Finally, the surgical site was examined to confirm that there were no intraoperative complications.

Conventional group: The operation procedure was the same as for the combined template group. However, rather than using the combined template, K‐wires were inserted under C‐arm fluoroscopy guidance, using lateral, inlet, and outlet views. Adjustments in the position of the K‐wire under fluoroscopy were made until the correct position was confirmed.

### *Evaluation Criteria*

#### *Operation Time*

Operation time is an important index to evaluate operation quality. In this study, the operative time is from the incision to the safe placement of screw.

#### *Radiation Exposure Times*

Radiation exposure can be harmful to humans, and intraoperative fluoroscopy should be minimized. The radiation exposure time refers to the number of c‐arm fluoroscopy exposures to confirm the safety of the screw placement in this study.

#### *Quality of the Reduction*

The quality of the reduction was used to evaluate postoperative fracture reduction, and it was measured by Matta score^8^ in this study, the maximum displacement of fracture reduction on radiographs was determined. A displacement of less than or equal to 4 mm was considered excellent, between 5 and 10 mm was regarded as good, between 10 and 20 mm was acceptable, and a displacement of more than 20 mm was regarded as poor.

#### *Grading Criteria*

The grading criteria was used to evaluate the safety of screws. According to Schep *et al*.^14^, the positions of the IS screws were graded based on the CT images: Class I, safe placement, screws solely in cancellous bone; Class II, safe placement but with screws touching cortical bone, such as the anterior sacral rim, nerve foramen, or spinal canal; and Class III, misplacement, screw penetration through cortical bone. Class I and Class II placements were considered successful and safe; Class III indicates the possibility of nerve damage.

#### *Difference between Actual and Simulated Screw Positions*

The difference between actual and simulated screw positions reflects the accuracy of the combined template, including the distance of the entry point and the angle deviation between the actual and simulated screws. Preoperative CT and postoperative CT were imported into MIMICS 21.0 (Materialize, Leuven, Belgium) for position registration, then the distance between the actual and simulated screws at the point of entrance and was measured and the angle deviation between the actual and simulated screws was measured. A smaller screw difference reflects higher accuracy of the combined template.

### *Statistical analysis*

All statistical analyses were performed in SPSS 19.0 (SPSS, Chicago, IL, USA). The* χ* ^*2*^‐test was used for discrete data between two groups, including gender, age distribution, trauma causes, Denis classification, Tile classification, number of screws, and follow‐up time; the Student *t*‐test was used for continuous data, including age, operation time and radiation exposure times; the Wilcoxon signed‐rank test was used for postoperative clinical indicators between two groups, including quality of the reduction and grading criteria. The confidence intervals was set as 95% and a *P*‐value less than 0.05 was considered to be statistically significant.

## Results

### *Patient Characteristics*

A total of 37 patients diagnosed with a sacral fracture and dislocation were included in this study from January 2016 to February 2018. All patients had sacral fractures due to an injury and instability of the posterior ring of the pelvis (type B and C fractures based on AO/OTA classification). There were no significant differences between the two groups in sex, age, trauma causes, Denis classification, Tile classification, and number of screws (*P* > 0.05) (Table 1).

**Table 1:** Clinical indicators of the study cohort and subgroups

Variables	All patients	Combined template group	Conventional group	Statistics	*P*‐value
Number of patients	37	19	18	—	—
Age (mean ± SD)	43 ± 9.8	43.1 ± 12.7	42.4 ± 5.7	0.206	0.838
Gender, *N* (%)					
Male	24	12	12	0.05	0.823
Female	13	7	6		
Age, *N* (%)					
<65	35	17	18	2.003	0.157
≥65	2	2	0		
Trauma causes, *N* (%)					
Motor vehicle accident	22	11	11	0.064	0.969
High‐energy fall	11	6	5		
Other injury	4	2	2		
Denis classification, *N* (%)					
Zone I fracture	13	7	6	0.05	0.823
Zone II fracture	24	12	12		
Tile classification, *N* (%)					
B	30	15	15	0.116	0.734
C	7	4	3		
Number of screws, *N* (%)					
S1	53	30	23	0.069	0.793
S2	20	12	8		
Follow‐up time, *N* (%)					
<6 months	7	3	4	0.249	0.618
≥6 months	30	16	14		

N, number; —, not included.

### *Follow‐up*

From the date of surgery, all patients were required to visit a specialist clinic once every 3 months. At each visit, the surgeon examined the surgical incision for any signs of wound infection. Radiographs and CT were also obtained at each follow‐up to confirm the fracture recovery. All patients were followed for 3 to 12 months, and no significant differences were found between the two groups (8.5 ± 3.2 months of follow up in the combined template group *vs* 8.3 ± 3.2 months of follow‐up in the conventional group, *P* > 0.05) (Table 1).

### *General results*

All patients underwent surgery successfully, and a total of 73 pedicle screws were implanted: 42 screws (30 S1, 12 S2) in 19 patients (combined template group) and 31 screws (23 S1, 8 S2) in 18 patients (conventional group). The average operative time of the combined template group for each screw was significantly less than that in the conventional group (25 ± 2.9 min *vs* 46.2 ± 9.6, *P* = 0.00). The radiation exposure times were significantly lower in the combined template group than that in the conventional group (12.1 ± 4 *vs* 56.1 ± 6.8, *P* = 0.00).

### *Screw evaluation*

**Figure 4:**
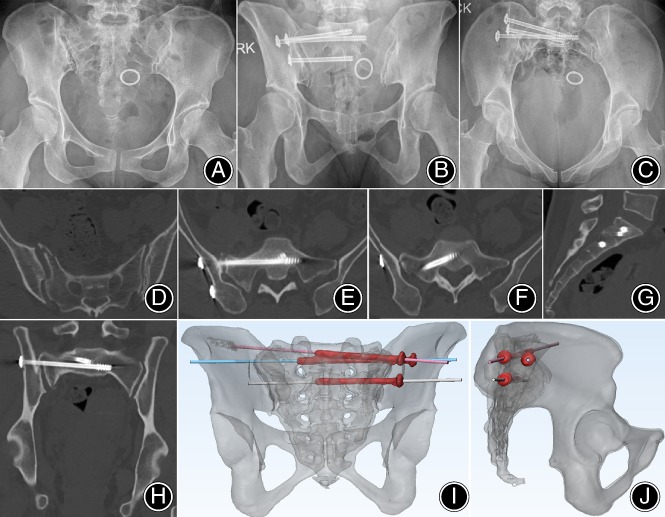
Preoperative and postoperative images of a 48‐year‐old female patient in the template group with sacral fracture and dislocation of the sacroiliac joint due to a traffic accident: (A) Preoperative anteroposterior radiograph images; (B, C) postoperative radiograph (outlet/inlet view); (D) preoperative CT axial image shows dislocation of the sacroiliac joint; (E–H) postoperative CT axial image confirming the placement of the IS screws; and (I) the deviation was measured on the superimposed images of the preoperative and postoperative 3D reconstructions.

The screw grading was superior in the combined template group than that in the conventional group (40 screws of grade I, 2 screws of grade II, and 0 screws of grade III in the combined template group *vs* 26 screws of grade I, 3 screws of grade II, and 2 screws of grade III in the conventional group, *P* = 0.031) (Table 2). In the combined template group, the mean distance of the entry point between the actual and virtual screws was 1.4 ± 0.9 mm, and the mean angle of deviation was 2.1° ± 1.6°(Fig. 4).

**Table 2:** Statistic analysis of intraoperative and postoperative clinical indicators of the two groups

Variables	All patients	Combined template group	Conventional group	Statistics	*P*‐value
Operation time per screw (min)	35 ± 12.8	25 ± 2.9	46.2 ± 9.6	−30.487	0.00*
Radiation exposure times (mean ± SD)	32 ± 23.6	12.1 ± 4	56.1 ± 6.8	−87.731	0.00*
Quality of the reduction, *N* (%)					
Excellent	11	6	5	−0.226	0.821
Good	22	11	11		
Fair	4	2	2		
Grading criteria, *N* (%)					
Grade I	66	40	26	−2.153	0.031*
Grade II	5	2	3		
Grade III	2	0	2		

* Statistical significance; *N*, number.

### *Complications*

In the conventional group, 1 patient developed symptoms of L5 nerve root stimulation immediately after the operation; the screw was removed 3 months after surgery and the symptoms were relieved.

## Discussion

### *General treatment for sacral fractures and iliosacral joint dislocation*

In the treatment of unstable sacral fractures, popular fixation methods include posterior percutaneous plate fixation, iliolumbar fixation, or IS screw fixation^15^. Posterior percutaneous tension plate fixation has the advantages of a simple operation, a short‐term learning curve, and less trauma. Kobbe *et al*.^16^ applied rear tension band steel plates to treat iliosacral joint dislocation and longitudinal fracture of the sacrum and achieved good clinical efficacy. This technique provides good horizontal stability of the posterior pelvis ring but without strengthening vertical stability between the pelvis and the spine. Papakostidis *et al*.^17^ found that the treatment of unstable sacral fractures with a posterior percutaneous plate could not help early weight‐bearing, with a failure rate of 17.3%. Iliolumbar fixation has excellent biomechanical properties^18^ and is very suitable for the treatment of complex sacral fractures. It transfers stress from the lumbar spine to the ilium and then to the acetabulum through connecting rods, helping patients with early weight‐bearing activities, but is associated with numerous complications, such as severe trauma and long recovery times. Internal fixation with IS screws for the treatment of unstable sacral fractures has been widely applied in the clinic. The procedure is not very traumatic, produces little blood loss, results in less soft tissue injury, has a low infection rate, and results in rapid fracture healing, among other advantages.

### *Difficulties of iliosacral screw insertion*

Although IS screws have many advantages in the treatment of sacral fractures or dislocations, IS screw placement assisted by traditional fluoroscopy has a higher incidence of vascular and nerve injuries^19^. Ozmeric *et al*.^20^ and Kim *et al*.^21^ applied special fluoroscopy positions to improve the accuracy of screw placement. The penetration of IS screws through the cortex could not be avoided^22^, ^23^, and the accuracy of screw placement could only be improved by increasing the operation and fluoroscopy times, which limits the efficiency of this technique. Computer navigation can reduce fluoroscopy time and improve the accuracy of screw placement, but the equipment is expensive. A 3D printed navigation template can assist IS screw implantation. The system includes the base and guide pipes, and individualized navigation templates are integrated structures. As such, this system has a number of disadvantages: (i) placement of the integrated guide template leads to a large incision in the skin; (ii) the length of the guide pipe changes the skin tension, thus affecting the accuracy of screw placement, especially for the S1 transverse screw; and (iii) for patients who need multi‐screw placement, the guide template cannot be removed after inserting the guide wire due to the inconsistent direction of each screw so that the K‐wires need to be replaced, which can affect the accuracy of the screw placement.

### *Advantages of combined template assisting iliosacral screw insertion*

In our study, the time required to place each screw in the combined template group was 25 ± 2.9 min; the total time of the fluoroscopy procedure was 12.1 ± 4, and the success rate of screw placement was 100% (42/42), which was significantly better than in the traditional group. Although much time was spent in the designing and printing of the combined template, the duration of the operation and the number of fluoroscopy exposures were significantly reduced. In our study, the penetration level of the external templates was significantly lower than that of traditional groups.

Ebraheim *et al*.^24^ described a method to evaluate the displacement between a simulated IS screw and an actual IS screw measured on sagittal CT at the screw tip, nerve root tunnel area, and entry point. The mean deviations of 2.2 ± 0.8 mm at the tip, 1.8 ± 1.6 mm at the nerve root tunnel, and 2.5 ± 1.8 mm at the entry point were reported by 3D CT perspective navigation registration. Takeba *et al*.^25^ adopted a similar approach; with the O‐arm navigation system, the average deviation of the screw entry point was 1.4 ± 0.9 mm. In this study, we reference the method of Takao, and our results are similar to those reported in their research. Among these similarities, computer navigation is used in both studies to guide screw placement. The accuracy of the IS trajectory planning was reflected in the small deviation between the planned and actual screw placement and the low cortical penetration rate. The trajectory was transfigured along the midline of the bone corridor, and the axial, coronal, and sagittal positions were confirmed.

### *Surgical tips*

We provide the following tips to improve the accuracy of screw placement based on the experience gained in this study.

(i) Adopt a thin layer for the printed model is suggested, generally less than 1 mm and with no interval scanning to improve the accuracy of the 3D model and, subsequently, the accuracy of the guide plate.

(ii) The surgeon should be involved in the design and simulation of the operation to minimize the base area where the guide plate can be fixed stably. This can also reduce the intraoperative dissection of soft tissue.

(iii) Preoperative reduction of the fracture and dislocation should be performed to improve the accuracy of screw placement.

(iv) The posterior superior iliac spine is a significant anatomical landmark that can be used as a reference point for preoperative design; the base of the combined template should primarily contain this area.

(v) The combined template should be firmly fixed during the operation to avoid deviation of screw placement caused by movement.

(vi) Multiple IS screws should be placed through S1 and S2 to significantly increase the fixation strength.

(vii) The guide pipe interface of the base can be cast as standard parts. The inner and outer guide pipes should be made of metal materials to reduce design time and cost and to improve mechanical stability.

### *Limitations*

This study has some limitations. First, some complications may be ignored during the short‐term follow up in our study. We will prolong the follow‐up time in later studies. Second, the patient sample size was relatively small in this study. However, a total of 73 screws were placed in this study, which was statistically significant for accuracy and safety analysis of screws. We will expand the sample size for further research. Finally, the safety and accuracy of IS screws were the focus of this study. The functional outcomes were not analyzed in this study and we will integrate the functional outcomes into research in future.

### *Conclusion*

In summary, the combined guiding template designed through 3D printing technology is a combined tool for IS insertion, which decreases the duration of radiation and of the operation, and avoids neurovascular injury caused by screw malpositioning. We achieved highly accurate and safe IS placement using this combined template. Further research on this technique deserves consideration.
